# Estimation of the cost of treatment by chemotherapy for early breast cancer in Morocco

**DOI:** 10.1186/1478-7547-8-16

**Published:** 2010-09-10

**Authors:** Saber Boutayeb, Abdesslam Boutayeb, Naoual Ahbeddou, Wiam Boutayeb, Essaadi Ismail, Mehdi Tazi, Hassan Errihani

**Affiliations:** 1 Service Oncologie Médicale, Institut National d'Oncologie, Université Mohamed V, Rabat, Morocco; 2 Department of Mathematics Faculty of Sciences, Boulevard Mohamed VI, BP: 717 Oujda, Morocco; 3 Unité Associée au CNRST URAC04, Boulevard Mohamed VI, BP: 717 Oujda, Morocco

## Abstract

**Background:**

Breast cancer is the first cancer in women both in incidence and mortality. The treatment of breast cancer benefited from the progress of chemotherapy and targeted therapies, but there was a parallel increase in treatment costs. Despite a relatively high incidence of many sites of cancer, so far, there is no national register for this disease in Morocco.

The main goal of this paper is to estimate the total cost of chemotherapy in the early stages of breast cancer due to its frequency and the chances of patients being cured. This study provides health decision-makers with a first estimate of costs and the opportunity to achieve the optimal use of available data to estimate the needs of antimitotics and trastuzumab in Morocco.

**Method:**

We start by evaluating the individual cost according to the therapeutic sub-groups, namely:

1. Patients needing chemotherapy with only anthracycline-based therapy.

2. Patients needing chemotherapy with both anthracycline and taxane but without trastuzumab.

3. Patients needing trastuzumab in addition to chemotherapy.

For each sub-group, the protocol of treatment is described, and the individual costs *per *unit, and for the whole cycle, are evaluated.

Then we estimate the number of women suffering from breast cancer on the basis of two data bases available in Morocco.

Finally, we calculate the total annual cost of treatment of breast cancer in Morocco.

**Results:**

The total cost of breast cancer in Morocco is given in Moroccan dirhams (MAD), the US dollar at the current exchange rate (MAD 10 = USD 1.30) and in international dollars or purchasing power parity (MAD 10 = PPP 1.95).

The cost of a therapy with trastuzumab is 8.4 times the cost of a sequential chemotherapy combining anthracycline and taxane, and nearly 60 times the cost of chemotherapy based on anthracycline alone.

Globally, between USD 13.3 million and USD 28.6 million need to be devoted every year by the Moroccan health authorities to treat women with localized breast cancer in keeping with international recommendations.

**Discussion:**

According to our estimation methods, the complete cost of adjuvant chemotherapy including trastuzumab will range from 1.3 to 2.4% of the global budget of the Moroccan Health Department (MAD 9.8 billion or USD 1.274 billion). Unfortunately, only one-third of the Moroccan population has healthcare insurance whereas for each patient the treatment with chemotherapy alone costs 1.15 times the annual minimum income (MAD 23,710 or USD 3,082), and treatment requiring both chemotherapy and trastuzumab costs 9.76 times the annual minimum income. For the tumour over expressing HER2Neu, we need to treat 25 women in order to save (cure) one woman: the calculated cost for one life saved is USD 663,000. The question is, is it cost-effective for an emerging country?

**Conclusion:**

In this paper we aimed at evaluating the total cost of chemotherapy in the early stages of breast cancer in order to provide health decision-makers with a first estimation and a good opportunity for the optimal use of available data for the needs of antimitotics and trastuzumab in Morocco. Different protocols were considered and the individual cost of the whole treatment was given according to therapies using anthracycline alone, sequential chemotherapy combining anthracycline and taxane, and sequential chemotherapy with trastuzumab. According to our estimations, Moroccan health authorities need to devote between USD 13.3 million and USD 28.6 million every year in order to treat women suffering from localized breast cancer in ways consistent with international recommended standards.

## Background

Cancer is one of the leading causes of mortality worldwide [1]. Morocco is an emerging country whose population approached 30 million in 2004, with about 10% of the people living in the region of Casablanca [2].

In Morocco, cancer is treated in five public centres and six private structures in addition to the cancer department of the military hospital. Despite a relatively high incidence of many sites of cancer across the country, so far there is no national register for this disease. In 2004, the first partial tentative provided a register of cancers in the region of Casablanca. A total of 5,414 cases of cancer were registered in this region but only 3,336 were living in the region, of whom 1,833 were women. In this region, of all sites of cancer, breast cancer was the most frequent representing 36.1%, followed by lung cancer (25.5%) and cervical cancer (12.6%) [2].

Worldwide, breast cancer is the first cancer in women both in incidence and mortality. It was also one of the first localization to benefit from the recent progress in systemic therapy especially targeted therapies [3]. Financing cancer treatment is a major challenge for both developed and developing countries, but developing countries are more vulnerable because of the limitations in their pharmaceutical industries and because of delayed diagnosis. Actually, several new drugs obtained approval for use in oncology. The common characteristic is, however, the high cost of this new generation of treatment.

The main objective of the present study is to provide an evaluation of the total cost of chemotherapy in the early stages of breast cancer due to the frequency of this cancer and the possibilities of cure. In the absence of registries and accurate data, the estimates yielded by this study will be of great importance to health decision-makers in Morocco, seeking to alleviate the disease burden in general and the cost of breast cancer in particular.

## Methods

The first step was the estimation of the individual cost according to the therapeutic sub-groups of breast cancer in curative situation (defined by absence of distant metastasis):

First of all, the different sub-groups of early breast cancer were defined according to international consensus criteria as indicated by the *Saint Gallen *and *Saint Paul de Vence *panel of experts as follows [4]:

1. Patients who don't need chemotherapy.

2. Patients needing chemotherapy

a. with only anthracycline-based therapy,

b. with both anthracycline and taxane but without trastuzumab,

c. with trastuzumab.

Second, for each sub-group, the protocol of treatment was described and for each protocol, we evaluated the individual cost *per *unit and whole cycle-set using for each drug the price of the cheapest generic as indicated by the *Agence Nationale de l'Assurance Maladie *which gives the average public price (APP) in Moroccan dirhams (MAD).

The second step was to evaluate the total number of women suffering from breast cancer. In the absence of a national register of cancer, two databases were used to estimate the total number of women with breast cancer:

1) A first database was provided by the annual public cancer centres treating patients with breast cancer. In this case, the total number of women suffering from breast cancer is obtained by adding the numbers of patients with localized breast cancer diagnosed in 2007 at the four public cancer centres in Morocco (Rabat, Casablanca, Agadir, and Oujda). This method is based on hospital cancer registries.

2) A second database was given by the regional register of cancer in Casablanca. In this case, the total number of women suffering from breast cancer is estimated by extrapolation, based on the assumption that the rate of breast cancer in Morocco is equivalent to the regional rate provided by the register of Casablanca (this source is a population-based cancer register, monitoring the frequency of cancer sub-types in the region of Casablanca by collecting case reports from different sources of clinicians and pathologists). It should be stressed that collected data are checked automatically to eliminate potential inaccuracies and duplicated cases.

The third step was to calculate the total cost of the treatment of breast cancer in curative situation in Morocco. In this stage, the percentages of patients assigned to each protocol were calculated in order to compute the total cost, with appropriate weights used for each treatment.

## Results

Estimation of the total cost of breast cancer treatment in Morocco is based on the estimation of individual treatment cost which, in turn, is based on the type of protocol applied. Women who need adjuvant chemotherapy may have one of three treatments: chemotherapy based on anthracycline without taxane [3,4], sequential chemotherapy combining anthracycline and taxane without trastuzumab [3,5] or sequential chemotherapy combining anthracycline, taxane and trastuzumab [5,6]. The details of each protocol are given in Additional file 1.

### 1. The cost of individual treatment (at the exchange rate MAD 10 = USD 1.3)

In order to evaluate the cost of individual treatment, we need the following information: 1) the unit price of different drugs used in adjuvant treatment of breast cancer, 2) the kind of therapy according to the sub-groups and the cost of a cycle for different protocols as indicated in the annex. In the interests of clarity and simplicity, we summarized this information in Table 1.

**Table 1:** Unit price for different drugs, cost of protocols by cycle and cost of individual whole treatment (values given in Moroccan dirhams and US dollars).

	Cost in MAD	Cost in USD
**Drug (presentation form)**	**Unit price**	**Unit price**

Doxorubicin (50 mg)	257	33.41

Cyclophosphamide (1000 mg)	56	7.28

Docetaxel (80 mg)	4,384	569.92

Trastuzumab (150 mg)	6,000	780.00

**Protocol**	**Cost by cycle**	**Cost by cycle**

AC60	650	84.50

Docetaxel	8,500	1,105.00

Trastuzumab	12,000	1,560.00

**Sub-group therapy**	**Individual cost of whole treatment**	**Individual cost of whole treatment**

Chemotherapy based on anthracycline without taxane	3,900	507.00

Sequential chemotherapy combining anthracycline and taxane	27,450	3,568.50

Sequential Chemotherapy and Trastuzumab (targeted therapy)	23,1450	30,088.50

The cost of a protocol with trastuzumab is nearly 20 times the cost of a protocol with AC60. For the individual whole treatment, the cost of a therapy with trastuzumab is 8.4 times the cost of a chemotherapy with anthracycline and taxane, and nearly 60 times the cost of a chemotherapy based on anthracycline alone.

The total cost by cycle was calculated for each protocol (for median body area of 1.6 m^2^) and given in the Moroccan currency and in US dollars. A protocol with trastuzumab is about 20 times the cost of a protocol with AC60. Similarly, for the individual whole treatment, the cost of a therapy with trastuzumab is 8.4 times the cost of a sequential chemotherapy combining anthracycline and taxane, and nearly 60 times the cost of a chemotherapy based on anthracycline alone.

### 2. Total recruitment of curative breast cancer in the public cancer centre network

As indicated earlier, the first database used to estimate the incidence of breast cancer and, consequently, its cost, was based on the annual public cancer centres' recruitment in breast cancer previously collected. In 2007, data were available from four centres as shown in Table 2. The National Institute of Oncology and the Centre in Casablanca are visited by patients from all regions of Morocco whereas the recently created centres in Agadir and Oujda remain regional.

**Table 2:** Curative breast cancer treated in different public cancer centres in Morocco.

National Institute of Oncology (NIO)	770
Centre of Casablanca	690

Regional Centre of Oujda	200

Regional Centre of Agadir	180

Global recruitment	1840

The two centres in Oujda and Agadir are regional centres recently created, whereas the two centres in Rabat and Casablanca are older and attract patients from all regions of Morocco.

### 3. Estimation of the incidence of curative breast cancer in Morocco

We used a second database to estimate the incidence of breast cancer in Morocco by extrapolating the rate of breast cancer provided by the regional register of cancer in Casablanca, to the whole country.

In 2004, the whole population of the Greater Casablanca region was 3,615,903 including 1,833,648 women and 1,782,255 men [2]. The global number of cancer patients registered was 5,414 including 3,336 cases originally from Casablanca [2]. Amongst 1,833 women diagnosed with cancer, 36.1% were breast cancer cases (662) [2]. Extrapolating these data to the whole country yields an estimation of 5,500 cases of breast cancer newly diagnosed in Morocco in 2004.

### 4. The percentages of patients assigned to each protocol

According to the statistics of the Moroccan cancer centres, about 75% of all newly diagnosed breast cancers belong to the curable category, approximately 4,125 patients out of 5,500 [7]. The number of patients needing chemotherapy represents 95% of all patients with localized disease (3,900 out of 4,125) with the following distribution [7]:

1) Patients who need anthracycline-based chemotherapy: 1,950 (50%)

2) Patients who need chemotherapy with both anthracycline and taxane: 1,950 (50%)

3) Patients who need trastuzumab in addition to anthracycline and taxane: 780 (20%).

### 5. The total cost of breast cancer treatment in Morocco

Finally, we end up with two estimations of the total cost of breast cancer treatment in Morocco. The first estimation is based on the database provided by the cancer centres' statistics (Estimate1, 2007). The second estimation was computed using the database given by the regional register of cancer in Casablanca (Estimate2, 2004). Table 3 summarizes the costs expressed in Moroccan dirhams (MAD), American dollars (USD) and international dollars (PPP).

**Table 3:** The total cost of breast cancer chemotherapy according to the cancer centres'statistics (2007) (Estimate1) and according to the estimated incidence of breast cancer in Morocco (2004) (Estimate2).

	Cost in MAD (×1000)		Cost in USD (×1000)		Cost in PPP (×1000)	
	
	Estimate1	Estimate2	Estimate1	Estimate2	Estimate1	Estimate2
Global cost of anthracycline- based therapy	3,432	7,605	446	989	669	1,483

Global cost of anthracycline and taxane based therapy	24,140	53,500	3,138	6,955	4,707	10,432

cost of trastuzumab	75,200	159,000	9,776	20,670	14,664	31,005

Totals	102,772	220,105	13,360	28,614	20,040	42,920

Between USD 13.3 and USD 28.6 millions need to be devoted every year by Moroccan health authorities to treat women with localized breast cancer according to international recommendations.

### 6. Cost per life saved by the addition of trastuzumab

Adjuvant trials have shown that the addition of trastuzumab to the chemotherapy improves the overall survival by 4%. For 25 women treated, one is cured by this addition [6]. Consequently, the cost for one life saved is the cost of treatment of 25 women treated by chemotherapy plus one year of trastuzumab, namely, USD 663,000.

## Discussion

Localized breast cancer is considered as curable whereas the existence of metastasis means a palliative strategy [3-5]. Usually, curative breast cancer is treated by a combination of surgery, radiotherapy, chemotherapy and hormone therapy (if positive hormone receptors) [3-5]. In this adjuvant setting, chemotherapy is indicated based on prognostic factors which are specified by consensus of scientific societies (ASCO, *Saint Gallen*, *Saint Paul de Vence*....) [3-5]. The addition of trastuzumab to chemotherapy depends on the over expression of the human epidermal growth factor receptor (HER 2 Neu). This receptor is targeted by the trastuzumab (monoclonal antibody) [5]. Indeed, well designed clinical trials in women with early breast cancer have demonstrated that one year's therapy with adjuvant intravenous trastuzumab (a loading dose followed by 6 mg/kg every 3 weeks or 2 mg/kg weekly) significantly improves disease-free survival and overall survival compared to observation (subsequent to chemotherapy) or chemotherapy alone in women with HER 2 Neu-positive disease [5].

According to international recommendations, Moroccan health authorities need to devote between USD 13.3 million and USD 28.6 million every year in order to treat women suffering from localized breast cancer. For the tumour over expressing HER 2 Neu, we need to treat 25 women in order to save (cure) one woman: the calculated cost for one life saved is USD 663,000. Is it cost-effective for an emerging country? This is a main question that we leave with health decision-makers.

It should be stressed that the first database includes only data from the public health system and excludes statistics of the five private centres which are not available. The cost is certainly higher than the USD 13.3 million *per *year estimated by this method. Conversely, the second method using the estimated incidence yielded a cost of USD 28.6 million *per *year but it assumes that all the new breast cancer cases are treated. Actually, only approximately one-third of the cancer cases in Morocco are treated.

According to our estimation methods, the complete cost of adjuvant chemotherapy including trastuzumab will represent between 1.3 and 2.4% of the global budget of the Health Department (MAD 9.8 billion or USD 1.274 billion). Unfortunately, only one-third of the Moroccan population has healthcare insurance whereas for each patient the treatment with chemotherapy alone costs 1.15 times the annual minimum income (MAD 23,710 or USD 3,082), and treatment needing both chemotherapy and trastuzumab costs 9.76 times the annual minimum income.

Comparison of the price of trastuzumab in different countries as reported in Table 4 shows that costs in Morocco are less than western countries but it is still expensive compared to income [8-10].

**Table 4:** Comparison of the price of trastuzumab in different countries

Country	Individual cost of trastuzumab in US dollars	Individual cost of trastuzumab. Equivalent in local currency
Morocco	USD 26,280	MAD 204,000

France	USD 39,629	EUR 27,594

United Kingdom	USD 41,247	GBP 25,866

USA	USD 70,000	USD 70,000

Australia	USD 44,146	AUD 50,000

Limitation of our study:

Some data used to calculate the number of patients candidate to chemotherapy like the ratio localized/metastatic and ratio of involvement of auxiliary lymph nodes are provided by local cancer centres' registers but are not published.

Using the second database to estimate the national incidence of breast cancer by extrapolating the data from the region of Casablanca to the whole country assumes that this regional incidence is the average incidence for the whole country. Such an assumption is valid under two conditions:

1. Either breast cancer incidence is nearly uniform through all regions, or

2. There is a compensating effect between regions with higher incidence and those with lower incidence than that of Casablanca.

A third method could be considered: using a weighted average of curative breast cancer treated in different centres taking into account the ratio incidence/regional population; but this method would have been biased since the National Institute of Oncology in Rabat and the Centre of Cancer in Casablanca attract patients from across the country.

## Conclusion

The burden of breast cancer is a major problem for both developed and developing countries; but citizens of developing countries are more vulnerable because of delayed diagnosis and lack of funds that allow for appropriate treatment, especially for women with very limited income. According to the figures released by the International Agency for Research on Cancer [11], breast cancer is the most frequent among all kinds of cancer in Morocco either by incidence (36.5%) or by mortality (19.7%). In the absence of national registries and accurate data, however, the country remains unable to adopt an optimal strategy that reduces as far as possible the burden of breast cancer under the constraints of limited resources.

In this paper we aimed to evaluate the global cost of chemotherapy in the early stages of breast cancer in order to provide health decision-makers with a first estimation and the opportunity to make the best use of available data for estimating the needs of antimitotics and trastuzumab in the country. Despite the limitations of our study as mentioned in the discussion section, we have estimated the cost of a cycle for different protocols. For the individual whole treatment, we found that the cost of a therapy with trastuzumab is 8.4 times the cost of a sequential chemotherapy combining anthracycline and taxane, and nearly 60 times the cost of a chemotherapy based on anthracycline alone. Nationwide, Moroccan health authorities need to devote between USD 13.3 million and USD 28.6 million every year in order to treat women suffering from localized breast cancer in ways that are consistent with international recommendations.

## Competing interests

The authors declare that they have no competing interests.

## Authors' contributions

BS, NA, MT, EI, and EH: Collected the data, interpreted results, and drafted sections of theManuscript.

BA and BW: designed analysis for this paper, conducted analysis.

All authors have read and approved the final version of the manuscript.

## Supplementary Material

Additional file 1 **Details of each protocol**. In this additional file, we give details of the sub-groups therapies and the type of protocol assigned to each sub-group of early breast cancer. Click here for file
