# Supplementary material for: Estimation of the cost of treatment by chemotherapy for early breast cancer in Morocco
Source: Cost Eff Resour Alloc. 2010 Sep 10;8:16. doi: 10.1186/1478-7547-8-16 (PMC2942794; doi:10.1186/1478-7547-8-16)
Supplement: Additional file 1 — Details of each protocol. In this additional file, we give details of the sub-groups therapies and the type of protocol assigned to each sub-group of early breast cancer. [file 1478-7547-8-16-S1.DOC]

**Estimation of the cost of treatment by chemotherapy for early breast cancer in Morocco**

**Saber Boutayeb**^1^**, Abdesslam Boutayeb**^2,3*^**, Naoual Ahbeddou**^1^**, Wiam Boutayeb², Ismail Essaadi**^1^**, Mehdi Tazi** ^1^ **and Hassan Errihani**^1^

^1^ Service Oncologie Médicale, Institut National d’Oncologie, Université Mohamed V, Rabat, Morocco

^2^Department of Mathematics Faculty of Sciences, Boulevard Mohamed VI, BP: 717 Oujda, Morocco

^3^ Unité Associée au CNRST URAC04, Boulevard Mohamed VI, BP: 717 Oujda, Morocco

^*^Corresponding author: Email: [x.boutayeb@menara.ma](mailto:x.boutayeb@menara.ma)

**Author’s mails:**

**BS:** [**boutayebdr@yahoo.fr**](mailto:boutayebdr@yahoo.fr)

**BA:** [**x.boutayeb@menara.ma**](mailto:x.boutayeb@menara.ma)

**NA:** [**lananoua@hotmail.fr**](mailto:lananoua@hotmail.fr)

**BW:** [**b.wiam@live.com**](http://mrd.mail.yahoo.com/compose?To=b.wiam@live.com)

**IE:** [**ismail_onco@yahoo.fr**](http://mrd.mail.yahoo.com/compose?To=ismail_onco@yahoo.fr)

**MT:** [**elmehditazi@yahoo.fr**](http://mrd.mail.yahoo.com/compose?To=elmehditazi@yahoo.fr)

**HE:** [**h_errihani@yahoo.fr**](mailto:h_errihani@yahoo.fr)

**Additional file1: Details of each protocol**

In this additional file, we give details of the sub-groups therapies and the type of protocol assigned to each sub-group of early breast cancer.

**Group I: Patients who don’t need chemotherapy.**

Patients belonging to this group are women with age greater than 35 years, having tumour of less than 2 cm without ganglion, responding to positive hormonal receptors but without amplification of HER 2 Neu.

**Group II: Patients who need adjuvant chemotherapy:**

1. Chemotherapy based on anthracycline without taxane:

This treatment is provided to patients not belonging to the “low risk” group but without over-expression of HER 2 Neu and without auxiliary lymph nodes invasion.

2. Sequential chemotherapy combining anthracycline and taxane, without trastuzumab:

This treatment is provided to patients not belonging to the “low risk” group without over-expression of HER 2 Neu but with invading auxiliary lymph nodes.

3. Sequential chemotherapy combining anthracycline, taxane and trastuzumab treatment:

Patients receiving this treatment are women not belonging to the “low risk” group, with over-expression of HER 2 Neu, with or without invading auxiliary lymph nodes.

The whole information concerning the subgroups and protocols with details is summarised in the table below.

**Table 5**: Sub-group therapies and protocols with details

| **Sub-group therapy** | **Protocols** | **Details of each protocol** |
| --- | --- | --- |
| Chemotherapy based on  anthracycline without  taxane | 6 cycles of AC60 | Adriamycine 60mg/m^2^  Cyclophosphamide 600mg/m^2^ |
| Sequential chemotherapy  combining anthracycline  and taxane | 3 cycles of AC60  followed by 3 cycles  of Docetaxel | Adriamycine 60mg/m^2^  Cyclophosphamide 600mg/m^2^  Docetaxel: 100 mg/ m^2^ |
| Sequential hemotherapy  and Trastuzumab  (targeted therapy) | 3 cycles of AC60  followed by 3 cycles  of Docetaxel  and Trastuzumab | Adriamycine 60mg/m^2^  Cyclophosphamide 600mg/m^2^  Docetaxel: 100 mg/ m^2^  trastuzumab: 8mg/kg (First injection) followed by 16 cycles of 6mg/ m^2^ three weekly (1 year treatment) |
